# Reproducibility of quantitative structural and physiological MRI measurements

**DOI:** 10.1002/brb3.759

**Published:** 2017-08-02

**Authors:** Stephen A. McGuire, S. Andrea Wijtenburg, Paul M. Sherman, Laura M. Rowland, Meghann Ryan, John H. Sladky, Peter V. Kochunov

**Affiliations:** ^1^ Aeromedical Research Department U.S. Air Force School of Aerospace Medicine Wright‐Patterson AFB Dayton OH USA; ^2^ Department of Neurology 59^th^ Medical Wing Joint Base San Antonio‐Lackland San Antonio TX USA; ^3^ Department of Neuroradiology 59^th^ Medical Wing Joint Base San Antonio‐Lackland San Antonio TX USA; ^4^ Maryland Psychiatric Research Center University of Maryland School of Medicine Baltimore MD USA

**Keywords:** diffusion tensor imaging, FLAIR, magnetic resonance spectroscopy, MRI consistency, MRI reproducibility, pseudocontinuous arterial spin labeling

## Abstract

**Introduction:**

Quantitative longitudinal magnetic resonance imaging and spectroscopy (MRI/S) is used to assess progress of brain disorders and treatment effects. Understanding the significance of MRI/S changes requires knowledge of the inherent technical and physiological consistency of these measurements. This longitudinal study examined the variance and reproducibility of commonly used quantitative MRI/S measurements in healthy subjects while controlling physiological and technical parameters.

**Methods:**

Twenty‐five subjects were imaged three times over 5 days on a Siemens 3T Verio scanner equipped with a 32‐channel phase array coil. Structural (T1, T2‐weighted, and diffusion‐weighted imaging) and physiological (pseudocontinuous arterial spin labeling, proton magnetic resonance spectroscopy) data were collected. Consistency of repeated images was evaluated with mean relative difference, mean coefficient of variation, and intraclass correlation (ICC). Finally, a “reproducibility rating” was calculated based on the number of subjects needed for a 3% and 10% difference.

**Results:**

Structural measurements generally demonstrated excellent reproducibility (ICCs 0.872–0.998) with a few exceptions. Moderate‐to‐low reproducibility was observed for fractional anisotropy measurements in fornix and corticospinal tracts, for cortical gray matter thickness in the entorhinal, insula, and medial orbitofrontal regions, and for the count of the periependymal hyperintensive white matter regions. The reproducibility of physiological measurements ranged from excellent for most of the magnetic resonance spectroscopy measurements to moderate for permeability‐diffusivity coefficients in cingulate gray matter to low for regional blood flow in gray and white matter.

**Discussion:**

This study demonstrates a high degree of longitudinal consistency across structural and physiological measurements in healthy subjects, defining the inherent variability in these commonly used sequences. Additionally, this study identifies those areas where caution should be exercised in interpretation. Understanding this variability can serve as the basis for interpretation of MRI/S data in the assessment of neurological disorders and treatment effects.

## 1 INTRODUCTION

Clinicians and scientists use longitudinal magnetic resonance imaging and spectroscopy (MRI/S) protocols to provide quantitative structural (T1‐ and T2‐weighted imaging) and physiological (microstructural properties of molecular diffusion, cerebral blood flow (CBF), and concentrations of neurochemicals) measurements to assess progression of neurological disorders and therapeutic effects of treatment. We quantified technical and normal physiological variability in commonly used MRI/S measurements to study the consistency of repeated measurements in healthy volunteers. Quantitative analysis of imaging and spectroscopy data was performed using standardized analysis pipelines. We minimized technical variability by utilizing a single MRI scanner and technician. Physiological variables were minimized by studying a select healthy population while restricting daily activities to a subject's consistent baseline and by imaging over a short interval.

Previous replication efforts in neuroimaging have reported scan‐to‐scan variability in the single modality measurements (Acheson et al., 2017; Dickerson et al., 2008; Han et al., 2006; Jovicich et al., 2014; Li et al., 2015; Maclaren, Han, Vos, Fischbein, & Bammer, 2014). We evaluated a battery of commonly used MRI sequences and measurements that ascertained both structural and physiological states of the brain in a well‐controlled group of healthy individuals. We report on the reproducibility and normal physiological variability for the state‐of‐art neuroimaging and spectroscopic measurements including reproducibility analysis for advanced bi‐exponential diffusion‐weighted imaging analysis. This included ascertainment of reproducibility of the cortical gray matter thickness, volume and number of hyperintensive white matter regions, resting CBF, fractional anisotropy of water diffusion, multi‐b‐value diffusion, and concentrations of important neurochemicals. The measurements included both gray‐matter‐ and white‐matter‐specific values providing an assessment of the normative tissue‐specific variance. Presenting reproducibility data collected under controlled physiological conditions while minimizing methodological variability may help planning of the future studies and performing power analyses of neuroimaging and spectroscopy measurements.

We selected three commonly used statistical metrics to provide a thorough assessment of reproducibility of MRI/S performed over a short interval in normal healthy volunteers. These metrics serve as the foundation for statistical inferences of the effects of disease or treatment on brain structure and/or physiology over time as measured by MRI/S. We used the variance observed across the three visits to perform a power analysis to calculate a hypothetical group size that is necessary to detect 3% and 10% group differences using a two‐tailed t‐test. This information should help to perform power analyses for the neuroimaging studies that utilize these measurements.

## 2 METHODS

### 2.1 Subjects

The study was reviewed and approved by the 59^th^ Medical Wing, United States Air Force (USAF), Institutional Review Board. Subjects were active duty members of the USAF recruited to serve as controls for an ongoing study on the effects of occupational exposure to extreme hypobaria in aircrew. All participants were recruited with strict adherence to the Department of Defense Instruction for Protection of Human Subjects (Department of Defense, 2011). For all subjects, participation was voluntary without commander involvement or knowledge. All subjects provided informed consent prior to participation. Subjects did not receive compensation for participation.

Twenty‐five (20 males/5 females, average age 25.8 ± 6.4 range 18–41 years) healthy subjects without hypertension, hyperlipidemia, or diabetes meeting USAF Flying Class III neurological standards were recruited (McGuire et al., 2014a,b). All subjects were in a military training environment with a consistently maintained meal time, sleep/wake time, and exercise program. Commencing 7 days prior to the first MRI and continuing throughout the study duration all subjects were alcohol free, drug/medication free, and tobacco free. Any new or acute illness was disqualifying. No subject was exposed to commercial air travel. To minimize diurnal physiological fluctuations, the daily time of repeat scans within the same subject was consistent for all three scans. All sequences were obtained during each MRI except for MRI#2, which did not include a fluid‐attenuated inversion recovery (FLAIR) sequence due to time constraints. Three subjects did not return for MRI#3.

### 2.2 Imaging methods

Imaging data were collected at the Wilford Hall Ambulatory Surgical Center, 59^th^ Medical Wing, Joint Base San Antonio‐Lackland, TX, using a Siemens 3T Verio scanner equipped with a 32‐channel phase array coil operated under quality control and assurance guidelines in accordance with recommendations by the American College of Radiology.

#### 2.2.1 Volumetric three‐dimensional FLAIR

Three‐dimensional FLAIR was utilized for analysis of white matter hyperintensities (WMH) as previously described (McGuire et al., 2014a,b). Briefly, FLAIR images were oriented to a common Talairach atlas‐based stereotactic frame using a nine‐parameter affine spatial transformation to ensure consistency of orientation for identification of the periependymal and subcortical regions (McGuire et al., 2013). The volumes of the FLAIR regions were calculated in the subject's frame by using an inverse of the spatial transformation. An experienced neuroanatomist blinded to the MRI study number manually traced WMH, while a neuroradiologist similarly blinded to the MRI study number provided MRI interpretation. Intrarater test–retest reproducibility was high (*r* = .95). For each lobe, we manually counted the number of WMH and used freely available Mango software version 4.0 (RRID:SCR_009603; http://ric.uthscsa.edu/Mango) to compute the total volume of WMH. WMH were divided into periependymal (adjacent to the ventricles) and subcortical (McGuire et al., 2013). Three‐dimensional imaging parameters were T1 magnetization‐prepared rapid gradient echo: repetition time (TR) = 2200 ms, echo time (TE) = 2.85 ms, isotropic resolution 0.80 mm, and FLAIR: TR = 4500 ms, TE = 1 ms, and isotropic resolution 1.00 mm. T1 imaging data were collected using motion‐corrected protocol where six individual segments were averaged following motion correction to improve signal‐to‐noise ratio (SNR) (Kochunov et al., 2006). The total T1 acquisition time was 18 min.

#### 2.2.2 Cortical gray matter thickness

The T1‐weighted (T1W) image processing for cortical gray matter thickness was conducted using the freely available FreeSurfer software version 5.3 (RRID:SCR_001847; http://surfer.nmr.mgh.harvard.edu/fswiki ^)^ and 10‐mm surface smoothing kernel. We used the freely available Enhanced Neuroimaging Genetics through Meta‐Analysis (ENIGMA; RRID:SCR_014649; http://enigma.ini.usc.edu/protocols/dti-protocols/) cortical gray matter thickness protocol that included visual quality assurance and control. The cortical gray matter thickness is measured as the Euclidian distance from the white matter mesh vertex to corresponding vertex on the cortical gray matter mesh. Cortical gray matter thickness measurements were averaged for individual cortical areas for both hemispheres; the whole‐brain cortical gray matter thickness measurement was obtained by averaging cortical gray matter thickness across left and right meshes. ENIGMA structural quality assurance/quality control (QA/QC) approach was used and one subject was excluded due to motion‐related artifacts.

#### 2.2.3 High angular resolution diffusion imaging

High angular resolution diffusion imaging (HARDI) was utilized for diffusion tensor imaging (DTI) and fractional anisotropy (FA) as previously reported. Briefly, DTI data were collected using a single‐shot echo‐planar, single refocusing spin‐echo, T2‐weighted sequence with a spatial resolution of 1.7 × 1.7 × 3.0 mm with sequence parameters of TE/TR = 87/8,000 ms, field of view (FOV) = 200 mm, axial slice orientation with 50 slices and no gaps, 64 isotropically distributed diffusion‐weighted directions, two diffusion weighting values (*b *=* *0 and 700 s/mm^2^), and five *b *=* *0 images. HARDI data for both groups were processed using the ENIGMA‐DTI (http://enigma.ini.usc.edu/protocols/dti-protocols/) pipeline (Jahanshad et al., 2013). ENIGMA‐DTI analysis pipeline is based on the tract‐based spatial statistics (TBSS) method, distributed as a part of FSL package (Smith et al., 2006). The ENIGMA‐DTI pipeline consists of a set of protocols and scripts to measure average whole‐brain FA value and average tract FA values for 10 major white matter tracts (corpus callosum, corticospinal, internal capsule, corona radiata, thalamic radiation, sagittal stratum, external capsule, cingulum, superior longitudinal fasciculus, and fronto‐occipital). ENIGMA‐DTI pipeline incorporates visual and quantitative quality assurance and control analyses. It includes visual inspection and two quantitative QA estimates: average motion and average projection distance. Prior research showed that FA estimates provided by this pipeline may become unstable if the average motion exceeds 2.5 mm and average projection distance exceeds 3.8 mm (Acheson et al., 2017). One DTI session was excluded from this analysis due to exceeding motion threshold.

#### 2.2.4 Multi‐b‐value diffusion imaging (MBI) protocol

The MBI protocol was developed based on q‐space protocols for in vivo mapping of water diffusion in the brain (Clark, Hedehus, & Moseley, 2002; Wu, Field, Whalen, & Alexander, 2011b; Wu et al., 2011a). This protocol consisted of 15 shells of b‐values (*b* = 250, 500, 600, 700, 800, 900, 1000, 1250, 1500, 1750, 2000, 2500, 3000, 3500, and 3800 s/mm^2^; diffusion gradient duration = 47 ms, diffusion gradient separation = 54 ms). Thirty isotropically distributed diffusion‐weighted directions were collected per shell, including 16 *b* = 0 images. The highest b‐value (*b* = 3,800 s/mm^2^) was chosen because the SNR for the corpus callosum in the average diffusion image (SNR = 6.1 ± 0.7) measured in five healthy volunteers (ages 25–50 years) during protocol development approached the empirically selected lower limit of SNR = 5.0. The *b*‐values and the number of directions per shell were chosen for improved fit of the bi‐exponential model and SNR (Jones, Horsfield, & Simmons, 1999). The imaging data were collected using a single‐shot, echo‐planar, single refocusing spin‐echo, T2‐weighted sequence with a spatial resolution of 1.7 × 1.7 × 4.6 mm and seven slices prescribed in sagittal orientation to sample the midsagittal band of the corpus callosum. The sequence control parameters were TE/TR = 120/1,500 ms with the FOV = 200 mm. The total scan time was about 10 min per subject.

The details of diffusivity‐permutability (PD) modeling are presented elsewhere (Kochunov, Chiappelli, & Hong, 2013). The PD model addresses a limitation of the standard DTI‐FA model, which assumes a single pool of anisotropically diffusing water. However, diffusion signal behaves as a biexponential function of *b*‐values, representing two, unrestricted and restricted, “pools” of water (*M*_u_ and *M*_r_, respectively) (Assaf et al., 2002, 2005; Clark et al., 2002; Wu et al., 2011a,b). Parameters derived from the biexponential modeling, such as perfusion‐diffusivity index (PDI), are therefore sensitive to membrane permeability (Kochunov et al., 2013, 2014; Sukstanskii, Ackerman, & Yablonskiy, 2003; Sukstanskii, Yablonskiy, & Ackerman, 2004). In short, diffusion images were preprocessed to perform an region of interest (ROI) based fit for a two‐compartment diffusion model (Equation (1)) that assumed that intravoxel signal is formed by a contribution from two compartments (Assaf et al., 2002, 2005; Clark et al., 2002; Panagiotaki et al., 2009; Wu et al., 2011a,b).

(1) $$S\left(b\right)=S_{0}\cdot \left(M_{u}\cdot e^{-bD_{u}}+\left(1-M_{u}\right)\cdot e^{-bD_{r}}\right)$$

(2) $$\text{PDI}=\frac{D_{r}}{D_{u}}$$

where *S*(*b*) is the average diffusion‐weighted signal for a given *b*‐value, averaged across all directions. *M*_u_ is the fraction of the signal that comes from the compartment with unrestricted diffusion. *M*_r_ (1 − *M*_u_) is the fraction of the signal that comes from the compartment with restricted diffusion. The PDI was calculated as the ratio of *D*_u_ and *D*_r_ (Equation (2)), which are the apparent diffusion coefficients of the unrestricted and restricted compartments, respectively. The diffusion‐weighted image for each of the *b*‐values *S*(*b*) was calculated for the four ROIs in cerebral white matter (the whole and the genu, body, and splenium of corpus callosum) and for the gray matter of the cingulate gyrus.

#### 2.2.5 Pseudocontinuous arterial spin labeling (pCASL) imaging

Pseudocontinuous arterial spin labeling (pCASL; RRID:SCR_015004) imaging data for gray and white matter were collected using gradient‐echo echo‐planar imaging with TE/TR = 16/4000 ms, 24 contiguous slices with 5 mm slice thickness, matrix = 64 × 64, 3.44 × 3.44 × 5.00 mm resolution (FOV = 220 mm) labeling gradient = 0.6 G/cm, bandwidth = 1594 Hz/pixel, 136 measurements, labeling offset = 90 mm, labeling duration = 2.1 s, and postlabeling delay = 0.93 s. In total, 68 alternating labeled and unlabeled image pairs were collected. Equilibrium magnetization (M0) images were collected using a long TR = 10‐s protocol. pCASL data were processed using the pipeline described elsewhere (University of South Carolina, 2012; http://www.mccauslandcenter.sc.edu/CRNL/tools/asl). Labeled and unlabeled pCASL images were independently motion corrected and a combined mean image was computed and coregistered to the spatially normalized T1W anatomical image. Perfusion‐weighted images were calculated by voxel‐wise subtractions of labeled and unlabeled images resulting in a mean perfusion‐weighted image. Absolute white matter perfusion or white matter CBF (blood flow and perfusion are interchangeable terms here) quantification was calculated in native space from the mean perfusion images. Voxel‐wise perfusion, in ml per 100 g/min, was calculated under the assumption that the postlabel delay was longer than average transfer time (Wang et al., 2002), where labeling efficiency was set at 0.99 and the mean transit time was set to 0.7 s based on empirical data. The data collection preceded the publication and was not based on the consensus guidelines for ASL‐in‐dementia parameters (Alsop et al., 2014). Instead, the imaging parameters were derived empirically to maximize detection of white matter perfusion by increasing labeling efficiency and signal‐to‐noise ratio. This was performed based on the methods described by others (van Gelderen, de Zwart, & Duyn, 2008; Wey, Wang, & Duong, 2012). In short, pCALS data in five healthy volunteers, representative of the study population (average age, 25.1 ± 6.4 range 20–35 years), were collected using the range of the labeling offset distances, labeling duration, and postlabeling delay times. Least‐square fitting was used to calculate the sequence parameters that maximized the labeling efficiency across cerebral white matter (WM) in all five subjects. This ensured that the derived parameters take into account the geometry of the MRI scanner and incorporate vascular physiology aspects of the subjects in this sample.

#### 2.2.6 Magnetic resonance spectroscopy

Proton magnetic resonance spectroscopy (MRS) data were acquired from voxels placed in frontal white matter and the anterior cingulate. For the frontal white matter region, short TE and long TE data were acquired using point resolved spectroscopy localization (TR = 1,500 ms, short TE = 30, long TE = 135 ms, number of signals averaged (NEX) = 256, 1.2‐kHz spectral width, 1,024 complex points, volume of interest (VOI) ~ 3.4 cm^3^). Data were acquired in both hemispheres and averaged together. For the anterior cingulate, the same short TE point resolved spectroscopy localization parameters were used with a voxel size of 6 cm^3^. A water reference (NEX = 8) was also acquired for all datasets to be used for phase and eddy current correction. A basis set of 19 metabolites was simulated using the gamma visual analysis (GAVA) software (Soher, Young, Bernstein, Aygula, & Maudsley, 2007) for use in quantifying the 30‐ms TE MRS data: alanine, aspartate, creatine (Cr), γ‐aminobuytric acid, glucose, glutamate (Glu), glutamine (Gln), glutathione (GSH), glycine, glycerophosphocholine, lactate, myo‐inositol (mI), N‐acetylaspartate (NAA), N‐acetylaspartylglutamate, phosphocholine, phosphocreatine, phosphoroylethanolamine, scyllo‐inositol, and taurine. A basis set of eight metabolites simulated using the same software package was generated for use in quantifying 135‐ms TE data: Cr, glycerophosphocholine, lactate, mI, NAA, N‐acetylaspartylglutamate, phosphocholine, and phosphocreatine. Each basis set was imported into LCModel (6.3‐0I) and used for quantification (Provencher, 2016). Metabolite levels were reported in institutional units, and all metabolites with percent standard deviation Cramer‐Rao lower bounds ≤20% were included in statistical analyses. One subject's MRI#1 and one subject's MRI#2 were excluded due to excessive artifact. As the anterior cingulate region is a mixture of gray and white matter, anterior cingulate metabolite levels were corrected for the proportion of the gray matter, white matter, and cerebrospinal fluid within the spectroscopic voxel using in‐house Matlab code based directly on the work of Gasparovic (Gasparovic et al., 2009). More specifically, tissue segmentation was performed in Statistical Parametric Mapping 8 (SPM8; Wellcome Trust Centre for Neuroimaging, 2015) using the T1W images acquired for voxel positioning to obtain the fraction of gray matter, white matter, and cerebrospinal fluid.

### 2.3 Statistical analysis

We used R‐Statistical Program (https://www.r-project.org/) and SPSS (IBM Corp., Armonk, NY) for data analysis. Mean and confidence intervals for each measure are found in Tables 1, 2, 3, 4, 5, 6, 7. To assess reproducibility, we examined the mean coefficient of variation (MCV, Equation (3)), the mean relative difference (MRD, Equation (4)), and the intraclass correlation (ICC, Equation (5)) to define the precision of measurement and reproducibility. MCV provides a general assessment of deviation relative to the mean as it is calculated as the standard deviation normalized by the average between visits. MRD provides information about the extremes of the data. The numerator is computed via the absolute difference between visits, resulting in only positive values, and then divided by the first visit value, thus computing a relative difference. ICC assesses the consistency of the variability among data across the three visits, and was calculated using a two‐way mixed model in SPSS MCV and MRD values closer to 0 are considered to be the most reproducible, and ICC values closer to 1 are considered more consistent and reproducible.

**Table 1:** Consistency of fluid‐attenuated inversion recovery (FLAIR)

FLAIR	V1 Mean [95% CI^a^]	V2 Mean [95% CI^a^]	MCV (%) [95% CI]	MRD (%) [95% CI]	ICC	Rating 3%^b^	Rating 10%^b^
Total volume	0.15 [0.12, 0.18]	0.14 [0.11, 0.17]	7.8 [4.9, 10.8]	10.4 [6.7, 14.1]	0.981	*N* = 35 (Moderate)	*N* = 4 (High)
Total lesions	4.82 [3.16, 6.48]	4.68 [3.04, 6.33]	7.1 [2.6, 11.6]	10.2 [3.7, 16.7]	0.989	*N* = 130 (Low)	*N* = 13 (High)
Subcortical volume	0.024 [0.011, 0.038]	0.025 [0.010, 0.040]	9.2 [4.2, 14.3]	14.0 [5.2, 22.9]	0.994	*N* = 38 (Moderate)	*N* = 5 (High)
Periependymal volume	0.13 [0.10, 0.15]	0.12 [0.095, 0.14]	9.1 [6.2, 12.0]	12.1 [8.5, 15.8]	0.974	*N* = 41 (Low)	*N* = 5 (High)
Subcortical number lesions	2.41 [0.78, 4.04]	2.36 [0.76, 4.00]	3.6 [−0.92, 8.2]	6.7 [−2.6, 16.1]	0.998	*N* = 39 (Moderate)	*N* = 5 (High)
Periependymal number lesions	2.41 [2.16, 2.66]	2.32 [2.08, 2.56]	9.5 [3.3, 15.7]	12.9 [4.6, 21.2]	0.465	*N* = 277 (Low)	*N* = 26 (High)

CI, confidence interval [lower limit, upper limit]; ICC, intraclass correlation; MRD, mean relative difference; MCV, mean coefficient of variation.

a *N* = 22.

b Reproducibility rating for 10% detection, power = 0.9, and significance = 0.05.

**Table 2:** Consistency of cortical thickness

Cortical thickness	V1 Mean [95% CI^a^]	V2 Mean [95% CI^a^]	V3 Mean [95% CI^b^]	MCV (%) [95% CI]	MRD (%) [95% CI]	ICC	Rating 3%^c^	Rating 10%^c^
Whole‐brain GM	2.67 [2.62, 2.71]	2.66 [2.62, 2.70]	2.67 [2.62, 2.71	0.80 [0.61, 0.98]	1.1 [0.80, 1.3]	0.980	*N* = 3 (High)	*N* = 2 (High)
*Left brain segments*								
lh mean thickness	2.67 [2.62, 2.72]	2.66 [2.62, 2.70]	2.67 [2.62, 2.71]	0.88 [0.64, 1.0]	1.2 [0.92, 1.4]	0.979	*N* = 3 (High)	*N* = 2 (High)
lh bankssts	2.64 [2.57, 2.71]	2.64 [2.59, 2.70]	2.64 [2.57, 2.70]	2.1 [1.6, 2.5]	2.7 [2.1, 3.2]	0.940	*N* = 11 (High)	*N* = 2 (High)
lh caudal anterior cingulate	2.77 [2.68, 2.86]	2.73 [2.63, 2.83]	2.74 [2.63, 2.84]	2.6 [1.9, 3.2]	3.3 [2.5, 4.1]	0.959	*N* = 17 (High)	*N* = 3 (High)
lh caudal middle frontal	2.74 [2.68, 2.79]	2.72 [2.67, 2.77]	2.71 [2.66, 2.77]	1.3 [1.0, 1.7]	1.7 [1.3, 2.1]	0.957	*N* = 5 (High)	*N* = 2 (High)
lh cuneus	1.99 [1.92, 2.06]	2.03 [1.96, 2.10]	2.02 [1.95, 2.09]	2.4 [1.7, 3.1]	3.2 [2.2, 4.2]	0.962	*N* = 13 (High)	*N* = 3 (High)
lh entorhinal	3.59 [3.49, 3.70]	3.51 [3.41, 3.62]	3.56 [3.46, 3.65]	3.9 [3.0, 4.8]	5.0 [3.9, 6.1]	0.747	*N* = 42 (Low)	*N* = 5 (High)
lh fusiform	2.88 [2.83, 2.94]	2.87 [2.81, 2.93]	2.87 [2.81, 2.93]	1.3 [1.0, 1.5]	1.6 [1.3, 1.9]	0.889	*N* = 5 (High)	*N* = 2 (High)
lh inferior parietal	2.63 [2.57, 2.68]	2.63 [2.58, 2.67]	2.64 [2.58, 2.70]	1.1 [0.85, 1.4]	1.5 [1.1, 1.8]	0.977	*N* = 4 (High)	*N* = 2 (High)
lh inferior temporal	3.01 [2.94, 3.07]	3.01 [2.95, 3.07]	3.01 [2.95, 3.08]	1.4 [1.1, 1.7]	1.8 [1.5, 2.2]	0.967	*N* = 6 (High)	*N* = 2 (High)
lh isthmus cingulate	2.54 [2.46, 2.61]	2.54 [2.46, 2.62]	2.51 [2.43, 2.60]	2.4 [2.0, 2.9]	3.1 [2.6, 3.7]	0.950	*N* = 15 (High)	*N* = 3 (High)
lh lateral occipital	2.28 [2.22, 2.33]	2.28 [2.22, 2.34]	2.30 [2.24, 2.36]	1.6 [1.1, 2.0]	2.1 [1.5, 2.7]	0.966	*N* = 7 (High)	*N* = 2 (High)
lh lateral orbitofrontal	2.92 [2.85, 2.98]	2.90 [2.83, 2.96]	2.87 [2.80, 2.95]	1.9 [1.5, 2.3]	2.4 [1.7, 3.0]	0.955	*N* = 9 (High)	*N* = 2 (High)
lh lingual	2.21 [2.15, 2.27]	2.21 [2.15, 2.27]	2.21 [2.15, 2.28]	1.8 [1.3, 2.3]	2.3 [1.7, 3.0]	0.974	*N* = 8 (High)	*N* = 2 (High)
lh medial orbitofrontal	2.72 [2.65, 2.79]	2.70 [2.63, 2.76]	2.68 [2.61, 2.75]	3.0 [2.5, 3.5]	3.9 [3.3, 4.5]	0.898	*N* = 21 (Moderate)	*N* = 3 (High)
lh middle temporal	3.10 [3.03, 3.17]	3.08 [3.01, 3.14]	3.10 [3.03, 3.16]	1.1 [0.78, 1.4]	1.4 [1.0, 1.8]	0.977	*N* = 4 (High)	*N* = 2 (High)
lh parahippocampal	2.83 [2.70, 2.96]	2.80 [2.67, 2.93]	2.80 [2.66, 2.93]	2.2 [1.9, 2.5]	2.8 [2.4, 3.3]	0.984	*N* = 13 (High)	*N* = 3 (High)
lh paracentral	2.53 [2.48, 2.59]	2.54 [2.49, 2.59]	2.56 [2.50, 2.62]	1.4 [1.1, 1.8]	1.8 [1.4, 2.2]	0.964	*N* = 6 (High)	*N* = 2 (High)
lh parsopercularis	2.85 [2.80, 2.91]	2.85 [2.80, 2.90]	2.85 [2.78, 2.91]	0.97 [0.74, 1.2]	1.3 [0.97, 1.5]	0.980	*N* = 3 (High)	*N* = 2 (High)
lh parsorbitalis	3.01 [2.94, 3.07]	2.98 [2.92, 3.04]	3.04 [2.97, 3.11]	1.8 [1.3, 2.4]	2.3 [1.6, 3.1]	0.963	*N* = 8 (High)	*N* = 2 (High)
lh parstriangularis	2.73 [2.67, 2.80]	2.71 [2.65, 2.77]	2.71 [2.64, 2.78]	1.3 [1.0, 1.6]	1.6 [1.3, 2.0]	0.981	*N* = 5 (High)	*N* = 2 (High)
lh pericalcarine	1.76 [1.69, 1.83]	1.80 [1.72, 1.88]	1.79 [1.72, 1.86]	2.7 [2.0, 3.4]	3.5 [2.6, 4.4]	0.978	*N* = 17 (High)	*N* = 3 (High)
lh postcentral	2.20 [2.15, 2.25]	2.20 [2.16, 2.25]	2.20 [2.15, 2.25]	1.2 [0.90, 1.5]	1.6 [1.2, 2.0]	0.972	*N* = 5 (High)	*N* = 2 (High)
lh posterior cingulate	2.60 [2.54, 2.65]	2.60 [2.54, 2.65]	2.58 [2.52, 2.64]	1.4 [1.1, 1.7]	1.9 [1.4, 2.3]	0.957	*N* = 6 (High)	*N* = 2 (High)
lh precentral	2.73 [2.69, 2.77]	2.72 [2.68, 2.75]	2.73 [2.69, 2.77]	1.0 [0.73, 1.3]	1.3 [0.96, 1.6]	0.951	*N* = 4 (High)	*N* = 2 (High)
lh precuneus	2.55 [2.49, 2.60]	2.54 [2.49, 2.60]	2.55 [2.50, 2.61]	0.98 [0.75, 1.2]	1.3 [0.97, 1.6]	0.983	*N* = 4 (High)	*N* = 2 (High)
lh rostral anterior cingulate	3.06 [2.95, 3.17]	3.02 [2.89, 3.15]	3.02 [2.89, 3.15]	3.0 [2.2, 3.9]	3.9 [2.8, 5.0]	0.952	*N* = 21 (Moderate)	*N* = 3 (High)
lh rostral middle frontal	2.62 [2.56, 2.69]	2.60 [2.54, 2.65]	2.59 [2.52, 2.66]	1.5 [1.2, 1.9]	2.0 [1.6, 2.4]	0.971	*N* = 6 (High)	*N* = 2 (High)
lh superior frontal	2.86 [2.81, 2.91]	2.84 [2.79, 2.88]	2.86 [2.82, 2.91]	1.6 [1.1, 2.1]	2.0 [1.4, 2.7]	0.918	*N* = 7 (High)	*N* = 2 (High)
lh superior parietal	2.30 [2.24, 2.35]	2.31 [2.26, 2.35]	2.31 [2.26, 2.36]	1.1 [0.84, 1.4]	1.4 [1.1, 1.7]	0.980	*N* = 4 (High)	*N* = 2 (High)
lh superior temporal	3.05 [2.99, 3.11]	3.05 [2.99, 3.11]	3.07 [3.00, 3.14]	1.2 [0.87, 1.5]	1.5 [1.1, 1.9]	0.970	*N* = 4 (High)	*N* = 2 (High)
lh supramarginal	2.75 [2.69, 2.81]	2.75 [2.70, 2.81]	2.75 [2.69, 2.82]	1.0 [0.83, 1.2]	1.3 [1.1, 1.6]	0.983	*N* = 4 (High)	*N* = 2 (High)
lh frontal pole	3.10 [2.99, 3.21]	3.12 [3.01, 3.24]	3.13 [3.01, 3.25]	3.2 [2.1, 4.3]	4.3 [2.8, 5.8]	0.918	*N* = 23 (Moderate)	*N* = 3 (High)
lh temporal pole	3.97 [3.88, 4.06]	3.97 [3.89, 4.06]	3.91 [3.81, 4.02]	2.2 [1.7, 2.8]	2.9 [2.2, 3.6]	0.919	*N* = 12 (High)	*N* = 2 (High)
lh transverse temporal	2.67 [2.57, 2.78]	2.70 [2.60, 2.79]	2.69 [2.60, 2.79]	2.0 [1.5, 2.6]	2.6 [2.0, 3.3]	0.971	*N* = 11 (High)	*N* = 2 (High)
lh insula	3.22 [3.15, 3.28]	3.24 [3.18, 3.30]	3.23 [3.17, 3.29]	2.2 [1.6, 2.8]	2.9 [2.1, 3.7]	0.890	*N* = 12 (High)	*N* = 2 (High)
*Right brain segments*								
rh mean thickness	2.67 [2.62, 2.71]	2.66 [2.62, 2.70]	2.66 [2.62, 2.71]	0.83 [0.64, 1.0]	1.1 [0.84, 1.4]	0.978	*N* = 3 (High)	*N* = 2 (High)
rh bankssts	2.81 [2.75, 2.87]	2.0 [2.75, 2.86]	2.76 [2.70, 2.82]	1.4 [1.2, 1.7]	1.9 [1.5, 2.2]	0.967	*N* = 6 (High)	*N* = 2 (High)
rh caudal anterior cingulate	2.66 [2.58, 2.74]	2.65 [2.59, 2.72]	2.67 [2.58, 2.75]	2.3 [1.9, 2.7]	3.0 [2.4, 3.5]	0.948	*N* = 14 (High)	*N* = 3 (High)
rh caudal middle frontal	2.65 [2.60, 2.70]	2.65 [2.60, 2.70]	2.64 [2.59, 2.69]	1.3 [0.99, 1.6]	1.7 [1.3, 2.1]	0.958	*N* = 5 (High)	*N* = 2 (High)
rh cuneus	2.03 [1.96, 2.09]	2.05 [1.98, 2.12]	2.03 [1.96, 2.11]	2.1 [1.4, 2.9]	2.7 [1.9, 3.6]	0.960	*N* = 11 (High)	*N* = 2 (High)
rh entorhinal	3.61 [3.52, 3.70]	3.56 [3.46, 3.66]	3.66 [3.55, 3.78]	4.2 [3.3, 5.1]	5.6 [4.3, 6.8]	0.781	*N* = 42 (Low)	*N* = 5 (High)
rh fusiform	2.89 [2.84, 2.94]	2.88 [2.83, 2.93]	2.86 [2.81, 2.92]	1.4 [1.1, 1.8]	1.8 [1.4, 2.3]	0.961	*N* = 6 (High)	*N* = 2 (High)
rh inferior parietal	2.66 [2.59, 2.72]	2.65 [2.60, 2.71]	2.66 [2.60, 2.73]	1.2 [0.95, 1.4]	1.5 [1.2, 1.8]	0.978	*N* = 5 (High)	*N* = 2 (High)
rh inferior temporal	3.04 [2.99, 3.10]	3.04 [2.97, 3.10]	3.03 [2.96, 3.10]	1.5 [1.1, 1.9]	1.9 [1.4, 2.4]	0.963	*N* = 6 (High)	*N* = 2 (High)
rh isthmus cingulate	2.44 [2.40, 2.49]	2.46 [2.40, 2.52]	2.45 [2.39, 2.51]	2.1 [1.6, 2.5]	2.7 [2.0, 3.3]	0.931	*N* = 11 (High)	*N* = 2 (High)
rh lateral occipital	2.31 [2.25, 2.38]	2.31 [2.25, 2.38]	2.33 [2.26, 2.40]	1.7 [1.3, 2.1]	2.2 [1.6, 2.8]	0.976	*N* = 8 (High)	*N* = 2 (High)
rh lateral orbitofrontal	2.88 [2.82, 2.94]	2.88 [2.83, 2.92]	2.85 [2.79, 2.92]	2.0 [1.6, 2.5]	2.6 [2.1, 3.2]	0.901	*N* = 11 (High)	*N* = 2 (High)
rh lingual	2.29 [2.24, 2.35]	2.31 [2.25, 2.38]	2.31 [2.25, 2.37]	1.6 [1.2, 2.0]	2.1 [1.6, 2.6]	0.977	*N* = 7 (High)	*N* = 2 (High)
rh medial orbitofrontal	2.77 [2.72, 2.83]	2.76 [2.69, 2.82]	2.73 [2.67, 2.79]	2.0 [1.5, 2.6]	2.6 [1.9, 3.3]	0.882	*N* = 12 (High)	*N* = 2 (High)
rh middle temporal	3.17 [3.11, 3.23]	3.15 [3.09, 3.21]	3.14 [3.07, 3.21]	1.3 [1.1, 1.6]	1.7 [1.4, 2.1]	0.972	*N* = 5 (High)	*N* = 2 (High)
rh parahippocampal	2.78 [2.67, 2.89]	2.76 [2.63, 2.89]	2.76 [2.63, 2.88]	2.2 [1.1, 2.8]	2.8 [2.1, 3.5]	0.980	*N* = 12 (High)	*N* = 2 (High)
rh paracentral	2.56 [2.50, 2.61]	2.57 [2.51, 2.62]	2.57 [2.51, 2.62]	1.4 [1.2, 1.7]	1.9 [1.5, 2.2]	0.961	*N* = 6 (High)	*N* = 2 (High)
rh parsopercularis	2.80 [2.75, 2.85]	2.80 [2.75, 2.85]	2.79 [2.74, 2.84]	1.0 [0.76, 1.3]	1.3 [0.97, 1.7]	0.971	*N* = 4 (High)	*N* = 2 (High)
rh parsorbitalis	3.02 [2.94, 3.10]	3.03 [2.97, 3.08]	3.02 [2.95, 3.08]	1.8 [1.3, 2.3]	2.4 [1.7, 3.0]	0.953	*N* = 9 (High)	*N* = 2 (High)
rh parstriangularis	2.73 [2.66, 2.80]	2.72 [2.65, 2.78]	2.71 [2.64, 2.78]	1.5 [1.2, 1.8]	2.0 [1.6, 2.4]	0.970	*N* = 6 (High)	*N* = 2 (High)
rh pericalcarine	1.77 [1.70, 1.84]	1.80 [1.72, 1.89]	1.79 [1.71, 1.86]	2.8 [2.2, 3.4]	3.7 [2.9, 4.5]	0.967	*N* = 18 (High)	*N* = 3 (High)
rh postcentral	2.17 [2.12, 2.22]	2.19 [2.13, 2.24]	2.16 [2.10, 2.21]	1.5 [0.81, 2.2]	1.9 [1.1, 2.7]	0.929	*N* = 7 (High)	*N* = 2 (High)
rh posterior cingulate	2.62 [2.55, 2.68]	2.61 [2.54, 2.68]	2.60 [2.55, 2.66]	1.3 [0.87, 1.7]	1.7 [1.2, 2.2]	0.962	*N* = 5 (High)	*N* = 2 (High)
rh precentral	2.69 [2.65, 2.73]	2.67 [2.63, 2.71]	2.68 [2.64, 2.72]	1.2 [0.70, 1.6]	1.5 [0.691, 2.0]	0.929	*N* = 4 (High)	*N* = 2 (High)
rh precuneus	2.54 [2.48, 2.59]	2.54 [2.48, 2.59]	2.54 [2.48, 2.60]	1.0 [0.78, 1.1]	1.3 [1.0, 1.5]	0.985	*N* = 4 (High)	*N* = 2 (High)
rh rostral anterior cingulate	3.12 [3.03, 3.21]	3.09 [3.00, 3.18]	3.09 [3.00, 3.19]	3.1 [2.3, 3.8]	3.9 [2.9, 4.8]	0.899	*N* = 23 (Moderate)	*N* = 3 (High)
rh rostral middle frontal		2.56 [2.52, 2.60]	2.56 [2.51, 2.60]	1.4 [1.1, 1.7]	1.8 [1.4, 2.2]	0.952	*N* = 5 (High)	*N* = 2 (High)
rh superior frontal	2.84 [2.78, 2.91]	2.82 [2.77, 2.87]	2.84 [2.78, 2.89]	1.7 [1.3, 2.1]	2.2 [1.7, 2.8]	0.937	*N* = 8 (High)	*N* = 2 (High)
rh superior parietal	2.32 [2.26, 2.37]	2.32 [2.27, 2.37]	2.32 [2.26, 2.37]	1.1 [0.92, 1.4]	1.5 [1.2, 1.8]	0.987	*N* = 4 (High)	*N* = 2 (High)
rh superior temporal	3.08 [3.01, 3.14]	3.07 [2.95, 3.19]	3.09 [3.02, 3.15]	1.1 [0.73, 1.4]	1.4 [0.95, 1.9]	0.980	*N* = 4 (High)	*N* = 2 (High)
rh supramarginal	2.74 [2.68, 2.80]	2.73 [2.68, 2.78]	2.72 [2.66, 2.79]	1.1 [0.83, 1.4]	1.4 [1.1, 1.8]	0.981	*N* = 4 (High)	*N* = 2 (High)
rh frontal pole	3.12 [2.99, 3.25]	3.07 [2.95, 3.19]	3.09 [2.96, 3.21]	3.2 [2.2, 4.1]	4.1 [2.8, 5.3]	0.940	*N* = 24 (Moderate)	*N* = 3 (High)
rh temporal pole	4.06 [3.96, 4.16]	4.01 [3.91, 4.11]	4.04 [3.93, 4.15]	2.3 [1.8, 2.9]	3.0 [2.3, 3.8]	0.921	*N* = 14 (High)	*N* = 3 (High)
rh transverse temporal	2.70 [2.61, 2.79]	2.71 [2.60, 2.81]	2.69 [2.59, 2.79]	2.3 [1.6, 3.0]	2.9 [2.1, 3.7]	0.969	*N* = 13 (High)	*N* = 3 (High)
rh insula	3.20 [3.14, 3.25]	3.15 [3.10, 3.20]	3.19 [3.13, 3.26]	2.4 [1.8, 2.9]	3.1 [2.4, 3.8]	0.809	*N* = 14 (High)	*N* = 3 (High)

CI, confidence interval [lower limit, upper limit]; MCV, mean coefficient of variation; ICC, intraclass correlation; MRD, mean relative difference; lh, left hemisphere; rh, right hemisphere; GM, gray matter.

a *N* = 25.

b *N* = 22.

c Reproducibility rating for 10% detection, power = 0.9, and significance = 0.05.

**Table 3:** Consistency of fractional anisotropy derived from diffusion tensor imaging

Fractional anisotropy	V1 Mean [95% CI^a^]	V2 Mean [95% CI^a^]	V3 Mean [95% CI^b^]	MCV (%) [95% CI]	MRD (%) [95% CI]	ICC	Rating 3%^c^	Rating 10%^c^
Average	0.50 [0.49, 0.51]	0.50 [0.49, 0.51]	0.50 [0.49, 0.51]	0.91 [0.51, 1.3]	1.2 [0.63,1.8]	0.979	*N* = 3 (High)	*N* = 2 (High)
Genu	0.74 [0.73, 0.76]	0.74 [0.73, 0.76]	0.75 [0.73, 0.76]	0.99 [0.69, 1.3]	1.3 [0.90, 1.7]	0.964	*N* = 4 (High)	*N* = 2 (High)
Body	0.73 [0.71, 0.74]	0.73 [0.71, 0.74]	0.73 [0.71,0.75]	1.6 [1.0, 2.1]	2.0 [1.3, 2.7]	0.965	*N* = 6 (High)	*N* = 2 (High)
Splenium	0.84 [0.83, 0.85]	0.84 [0.83,0.85]	0.84 [0.82, 0.85]	0.88 [0.58, 1.1]	1.1 [0.76,1.5]	0.969	*N* = 3 (High)	*N* = 2 (High)
Fornix	0.55 [0.53, 0.56]	0.55 [0.54, 0.57]	0.55 [0.53, 0.57]	3.8 [2.5, 5.2]	5.0 [3.1, 6.9]	0.865	*N* = 30 (Moderate)	*N* = 4 (High)
Corticospinal	0.70 [0.68, 0.72]	0.71 [0.69, 0.73]	0.71 [0.69, 0.72]	2.8 [1.6, 3.9]	3.8 [2.0, 5.5]	0.910	*N* = 21 (Moderate)	*N* = 3 (High)
Internal capsule	0.68 [0.67, 0.69]	0.68 [0.67, 0.70]	0.68 [0.67, 0.70]	1.6 [0.86, 2.2]	2.1 [1.1, 3.1]	0.942	*N* = 5 (High)	*N* = 2 (High)
Corona radiata	0.53 [0.52, 0.54]	0.53 [0.52, 0.54]	0.53 [0.52, 0.54]	1.5 [1.0, 1.9]	1.9 [1.3, 2.6]	0.967	*N* = 5 (High)	*N* = 2 (High)
Thalamic radiation	0.67 [0.66, 0.68]	0.67 [0.66, 0.68]	0.67 [0.65, 0.68]	1.2 [0.77, 1.7]	1.6 [0.96, 2.2]	0.960	*N* = 4 (High)	*N* = 2 (High)
Sagittal striatum	0.61 [0.60, 0.62]	0.61 [0.60, 0.63]	0.61 [0.59, 0.62]	1.6 [1.1, 2.1]	2.1 [1.4, 3.3]	0.965	*N* = 5 (High)	*N* = 2 (High)
External capsule	0.54 [0.53, 0.55]	0.54 [0.53, 0.56]	0.54 [0.53, 0.55]	1.8 [1.2, 2.4]	2.4 [1.5, 3.3]	0.934	*N* = 7 (High)	*N* = 2 (High)
Cingulum	0.68 [0.67, 0.70]	0.69 [0.67, 0.70]	0.69 [0.67, 0.71]	2.0 [1.4, 2.6]	2.7 [1.7,3.6]	0.955	*N* = 9 (High)	*N* = 2 (High)
Superior longitudinal fasciculus	0.55 [0.54, 0.56]	0.55 [0.54, 0.56]	0.55 [0.54, 0.56]	1.7 [1.3, 2.2]	2.3 [1.6, 3.0]	0.952	*N* = 7 (High)	*N* = 2 (High)
Fronto‐occipital	0.59 [0.58, 0.60]	0.59 [0.58, 0.61]	0.59 [0.58, 0.61]	2.5 [1.6, 3.4]	3.4 [2.0, 4.7]	0.882	*N* = 12 (High)	*N* = 2 (High)
Superior fronto‐occipital	0.59 [0.58, 0.61]	0.60 [0.58, 0.62]	0.60 [0.59, 0.62]	3.3 [2.3, 4.4]	4.5 [2.9, 6.1]	0.874	*N* = 21 (Moderate)	*N* = 3 (High)
Inferior fronto‐occipital	0.58 [0.57, 0.60]	0.59 [0.57, 0.60]	0.59 [0.57, 0.60]	2.4 [1.5, 3.2]	3.2 [2.0, 4.3]	0.917	*N* = 13 (High)	*N* = 3 (High)

CI, confidence interval [lower limit, upper limit]; ICC, intraclass correlation; MRD, mean relative difference; MCV, mean coefficient of variation.

a *N* = 25.

b *N* = 21 (three subjects only completed two visits and one dataset removed due to artifact.

c Reproducibility rating for 10% detection, power = 0.9, and significance = 0.05.

**Table 4:** Consistency of multi‐*b*‐value diffusion imaging (MBI or q‐space) for corpus callosum and anterior cingulate

q‐space	V1 Mean [95% CI^a^]	V2 Mean [95% CI^a^]	V3 Mean [95% CI^b^]	MCV (%) [95% CI]	MRD (%) [95% CI]	ICC	Rating 3%^c^	Rating 10%^c^
*Corpus callosum (CC)*								
CC *M*_u_	0.53 [0.51, 0.54]	0.52 [0.51, 0.54]	0.52 [0.51, 0.54]	2.1 [1.5, 2.7]	2.6 [1.8, 4.4]	0.967	*N* = 11 (High)	*N* = 2 (High)
CC PDI	0.037 [0.034, 0.041]	0.038 [0.034, 0.042]	0.038 [0.033, 0.042]	11.1 [7.8, 14.4]	13.8 [9.2, 23.0]	0.911	*N* = 28 (Moderate)	*N* = 4 (High)
*Anterior cingulate (Cing)*								
Cing *M*_u_	0.67 [0.66, 0.69]	0.66 [0.65, 0.68]	0.68 [0.66, 0.69]	4.0 [2.9, 5.1]	5.2 [3.8, 6.7]	0.434	*N* = 37 (Moderate)	*N* = 4 (High)
Cing PDI	0.047 [0.036, 0.058]	0.050 [0.040, 0.060]	0.040 [0.031, 0.049]	29.8 [21.3, 38.4]	39.8 [26.1, 53.8]	0.646	*N* = 240 (Low)	*N* = 2 (Moderate)

CI, confidence interval [lower limit, upper limit]; MCV, mean coefficient of variation; ICC, intraclass correlation; MRD, mean relative difference; *M*_u,_ unrestricted water component; PDI, perfusion‐diffusivity index.

a *N* = 25.

b *N* = 22 (three subjects completed only two visits).

c Reproducibility rating for 10% detection, power = 0.9, and significance = 0.05.

**Table 5:** Consistency of GM blood flow as measured by pCASL

pCASL GM	V1 Mean [95% CI^a^]	V2 Mean [95% CI^a^]	V3 Mean [95% CI^b^]	MCV (%) [95% CI]	MRD (%) [95% CI]	ICC	Rating 3%^c^	Rating 10%^c^
Average CBF	51.8 [48.2, 55.4]	52.8 [49.1, 56.5]	50.9 [47.3, 54.6]	4.5 [3.3, 5.8]	5.8 [4.2, 7.4]	0.971	*N* = 45 (Low)	*N* = 5 (High)
Frontal pole	58.8 [54.6, 62.9]	59.0 [54.8, 63.2]	56.5 [52.2, 61.0]	6.6 [5.0,8.2]	8.5 [6.3, 10.7]	0.954	*N* = 95 (Low)	*N* = 10 (High)
Insular cortex	45.7 [42.3, 49.1]	46.6 [43.3, 50.0]	43.7 [40.2, 47.3]	7.5 [6.0, 9.0]	9.6 [7.6, 11.6]	0.931	*N* = 124 (Low)	*N* = 12 (High)
Superior frontal gyrus	68.7 [63.3, 74.1]	70.6 [65.4, 75.8]	67.3 [62.2, 72.5]	5.7 [4.2, 7.2]	7.5 [5.4, 9.6]	0.965	*N* = 69 (Low)	*N* = 7 (High)
Middle frontal gyrus	67.7 [62.9, 72.5]	69.4 [64.5, 74.3]	65.6 [60.4, 70.9]	6.4 [5.1, 7.8]	8.3 [6.4, 10.2]	0.957	*N* = 91 (Low)	*N* = 9 (High)
Inferior frontal gyrus parstriangularis	55.1 [51.4, 58.9]	56.7 [52.6, 60.8]	53.1 [48.9, 57.3]	7.1 [5.6, 8.6]	9.2 [7.2, 11.1]	0.939	*N* = 115 (Low)	*N* = 11 (High)
Inferior frontal gyrus parsopercularis	56.5 [52.7, 60.3]	57.7 [53.8, 61.6]	55.2 [51.1, 59.4]	7.0 [5.7, 8.3]	8.9 [7.2, 10.7]	0.934	*N* = 109 (Low)	*N* = 11 (High)
Precenteral gyrus	66.3 [61.5, 71.0]	68.4 [63.3, 73.4]	65.4 [60.6, 70.2]	5.6 [4.4, 7.0]	7.4 [5.6, 9.1]	0.960	*N* = 73 (Low)	*N* = 8 (High)
Temporal pole	42.1 [39.0, 45.2]	44.0 [40.8, 47.3]	40.4 [36.9, 43.8]	8.7 [7.0,10.4]	11.1 [9.0, 13.2]	0.926	*N* = 167 (Low)	*N* = 16 (High)
Superior temporal gyrus, anterior division	46.8 [42.9, 50.8]	47.3 [43.6, 50.9]	43.1 [39.7, 46.6]	9.1 [7.1, 11.1]	11.5 [9.1, 13.9]	0.901	*N* = 192 (Low)	*N* = 18 (High)
Superior temporal gyrus, posterior division	53.6 [49.2, 58.0]	53.0 [48.9, 57.2]	50.2 [46.2, 54.2]	7.9 [6.3, 9.5]	10.1 [8.0, 12.1]	0.929	*N* = 142 (Low)	*N* = 14 (High)
Middle temporal gyrus, anterior division	41.2 [38.1, 44.3]	42.7 [39.5, 46.0]	38.7 [35.1, 42.2]	8.9 [7.2 10.7]	11.4 [9.2, 13.5]	0.931	*N* = 175 (Low)	*N* = 17 (High)
Middle temporal gyrus, posterior division	46.7 [43.1, 50.4]	47.5 [43.7, 51.2]	43.9 [40.1, 47.7]	8.3 [6.6, 10.0]	10.5 [8.4, 12.7]	0.929	*N* = 156 (Low)	*N* = 15 (High)
Middle temporal gyrus, temporo‐occipital part	47.2 [43.7, 50.7]	48.5 [44.4, 52.6]	46.0 [42.2, 49.7]	7.6 [6.0, 9.2]	9.9 [7.8, 12.0]	0.925	*N* = 136 (Low)	*N* = 13 (High)
Inferior temporal gyrus, anterior division	36.2 [33.0, 39.4]	37.1 [33.9, 40.2]	34.1 [30.7, 37.5]	10.1 [7.7, 12.6]	12.7 [10.0, 15.5]	0.897	*N* = 230 (Low)	*N* = 22 (Moderate)
Inferior temporal gyrus, posterior division	44.1 [40.5, 47.8]	45.2 [41.5, 48.9]	41.9 [37.7, 46.0]	9.2 [6.9, 11.6]	11.3 [9.0, 14.6]	0.926	*N* = 188 (Low)	*N* = 18 (High)
Inferior temporal gyrus, temporo‐occipital part	43.3 [40.1, 46.5]	44.5 [40.9, 48.2]	41.7 [38.4, 45.1]	8.2 [6.5, 10.0]	10.7 [8.3, 12.8]	0.916	*N* = 152 (Low)	*N* = 15 (High)
Postcentral gyrus	64.0 [59.2, 68.9]	66.4 [61.4, 71.4]	64.4 [59.7, 69.1]	5.6 [4.3, 6.9]	7.6 [5.6, 9.2]	0.962	*N* = 71 (Low)	*N* = 7 (High)
Superior parietal lobule	62.2 [57.1, 67.3]	64.1 [58.5, 69.7]	62.6 [57.0, 68.2]	5.6 [4.6, 6.6]	7.4 [5.9, 8.7]	0.973	*N* = 72 (Low)	*N* = 7 (High)
Supramarginal gyrus, anterior division	55.4 [51.5, 59.4]	57.0 [53.0, 61.1]	55.9 [52.1, 59.7]	6.5 [5.1, 7.8]	8.8 [6.6, 10.3]	0.941	*N* = 90 (Low)	*N* = 9 (High)
Supramarginal gyrus, posterior division	55.8 [51.9, 59.4]	56.4 [52.3, 60.6]	54.8 [51.0, 58.7]	5.9 [4.6, 7.1]	7.6 [6.0, 9.4]	0.953	*N* = 77 (Low)	*N* = 8 (High)
Angular gyrus	55.6 [51.8, 59.5]	57.0 [52.8, 61.2]	55.7 [51.9, 59.6]	5.8 [4.4, 7.2]	7.8 [5.8, 9.8]	0.947	*N* = 77 (Low)	*N* = 8 (High)
Lateral occipital cortex, superior division	56.3 [52.0, 60.6]	58.5 [53.6, 63.4]	57.8 [52.9, 62.7]	5.7 [4.4, 7.0]	7.5 [5.6, 9.2]	0.962	*N* = 76 (Low)	*N* = 8 (High)
Lateral occipital cortex, inferior division	44.3 [40.6, 47.9]	45.8 [41.5, 50.1]	44.4 [40.5, 48.4]	7.8 [6.0, 9.5]	10.1 [7.8, 12.5]	0.930	*N* = 147 (Low)	*N* = 14 (High)
Intracalcarine cortex	40.0 [36.6, 43.4]	41.5 [37.6, 45.4]	40.2 [36.1, 44.4]	8.1 [6.3, 10.0]	10.7 [8.1, 13.3]	0.927	*N* = 162 (Low)	*N* = 15 (High)
Frontal medial cortex	50.4 [46.1, 54.7]	51.8 [48.2, 55.4]	48.3 [44.0, 52.5]	9.2 [7.2, 11.2]	12.0 [9.0, 15.0]	0.922	*N* = 193 (Low)	*N* = 18 (High)
Juxtapositional lobule cortex	69.5 [64.2, 74.8]	71.1 [66.3, 75.9]	67.7 [63.1, 72.3]	6.2 [4.9, 7.6]	8.1 [6.3, 10.1]	0.948	*N* = 90 (Low)	*N* = 9 (High)
Subcallosal cortex	52.0 [47.8, 56.1]	53.1 [48.4, 57.7]	48.7 [44.1, 53.3]	11.6 [9.5, 13.4]	14.1 [12.1, 18.1]	0.896	*N* = 303 (Low)	*N* = 28 (Moderate)
Paracingulate gyrus	57.5 [53.1, 61.9]	58.4 [54.4, 62.5]	54.7 [50.1, 59.4]	7.4 [5.8, 9.1]	9.4 [7.3, 11.5]	0.940	*N* = 118 (Low)	*N* = 12 (High)
Cingulate gyrus, anterior division	56.0 [51.6, 60.4]	57.2 [53.6, 60.8]	54.5 [50.6, 58.4]	8.3 [6.6, 10.0]	10.8 [8.4, 13.2]	0.893	*N* = 155 (Low)	*N* = 15 (High)
Cingulate gyrus, posterior division	62.3 [58.2, 66.3]	63.7 [59.1, 68.3]	62.1 [57.4, 66.9]	5.7 [4.4, 7.1]	7.5 [5.6, 9.3]	0.947	*N* = 75 (Low)	*N* = 8 (High)
Precuneous cortex	55.7 [51.6, 59.8]	58.2 [53.4, 62.9]	57.6 [52.7, 62.5]	5.9 [4.6, 7.2]	7.7 [6.0, 9.5]	0.959	*N* = 83 (Low)	*N* = 8 (High)
Cuneal cortex	46.2 [42.4, 50.1]	47.7 [43.3, 52.0]	48.5 [43.6, 53.3]	7.0 [5.2, 8.8]	9.1 [6.6, 11.6]	0.946	*N* = 119 (Low)	*N* = 12 (High)
Frontal orbital cortex	47.6 [44.2, 51.0]	49.4 [45.6, 53.1]	45.3 [41.3, 49.2]	8.0 [6.4, 9.7]	10.2 [8.1, 12.4]	0.947	*N* = 140 (Low)	*N* = 14 (High)
Parahippocampal gyrus, anterior division	37.4 [34.4, 40.4]	38.3 [35.0, 41.7]	35.9 [32.3, 39.5]	9.6 [7.2, 12.0]	12.2 [9.1, 15.2]	0.888	*N* = 206 (Low)	*N* = 19 (High)
Parahippocampal gyrus, posterior division	32.7 [30.6, 34.9]	33.8 [31.2, 36.4]	31.9 [29.4, 34.4]	6.9 [5.2, 8.7]	9.2 [6.7, 11.6]	0.900	*N* = 118 (Low)	*N* = 12 (High)
Lingual gyrus	36.7 [33.8, 39.6]	38.2 [35.0, 41.5]	36.7 [33.4, 40.1]	8.2 [6.4, 10.0]	10.8 [8.3, 13.3]	0.920	*N* = 159 (Low)	*N* = 15 (High)
Temporal fusiform cortex, anterior division	33.9 [30.8, 37.0]	34.7 [31.7, 37.7]	31.4 [28.1, 34.7]	9.9 [6.9, 12.8]	12.3 [8.9, 15.7]	0.885	*N* = 237 (Low)	*N* = 22 (Moderate)
Temporal fusiform cortex, posterior division	33.2 [30.8, 35.5]	33.4 [31.0, 35.8]	31.5 [28.8, 34.2]	7.2 [5.5, 9.0]	9.1 [7.0, 11.3]	0.926	*N* = 116 (Low)	*N* = 11 (High)
Temporal occipital fusiform cortex	33.2 [30.7, 35.6]	33.4 [30.5, 36.3]	32.0 [29.0, 35.0]	8.5 [6.7, 10.3]	10.8 [8.5, 13.1]	0.909	*N* = 169 (Low)	*N* = 16 (High)
Occipital fusiform gyrus	34.5 [31.7, 37.4]	36.2 [32.7, 39.7]	33.9 [30.5, 37.3]	8.0 [6.5, 9.6]	10.4 [8.3, 12.5]	0.937	*N* = 150 (Low)	*N* = 14 (High)
Frontal operculum cortex	48.4 [44.8, 52.0]	49.4 [45.9, 52.9]	45.9 [41.9, 50.0]	7.9 [6.4, 9.4]	10.0 [8.2, 11.9]	0.934	*N* = 141 (Low)	*N* = 14 (High)
Central opercular cortex	46.2 [43.0, 49.4]	47.7 [44.6, 50.8]	44.7 [41.6, 50.0]	7.4 [6.0, 8.9]	9.7 [7.7, 11.6]	0.907	*N* = 128 (Low)	*N* = 12 (High)
Parietal operculum cortex	48.3 [45.1, 51.5]	48.5 [45.0, 52.0]	46.6 [43.2, 50.0]	7.8 [6.5, 9.1]	10.0 [8.4, 11.6]	0.907	*N* = 139 (Low)	*N* = 13 (High)
Planum polare	48.2 [44.7, 51.7]	47.5 [44.0, 51.0]	43.7 [40.3, 47.1]	9.3 [8.0, 10.7]	11.8 [10.1, 13.5]	0.914	*N* = 197 (Low)	*N* = 19 (High)
Heschl's gyrus	55.5 [50.8, 58.8]	54.9 [50.4, 59.4]	52.3 [48.0, 56.7]	7.9 [6.3, 9.4]	10.1 [8.1, 12.0]	0.930	*N* = 147 (Low)	*N* = 14 (High)
Planum temporale	54.7 [50.5, 58.8]	54.1 [49.8, 58.4]	52.2 [48.1 56.2]	7.5 [5.9, 9.1]	9.6 [7.5, 11.7]	0.925	*N* = 129 (Low)	*N* = 13 (High)
Supracalcarine cortex	42.9 [39.3, 46.5]	43.9 [39.7, 48.1]	43.2 [38.8, 47.6]	8.5 [6.4, 10.5]	11.0 [8.1, 13.9]	0.917	*N* = 174 (Low)	*N* = 17 (High)
Occipital pole	43.9 [40.2, 47.7]	44.8 [40.3, 49.4]	44.0 [39.6, 48.4]	9.8 [7.7, 12.0]	12.7 [9.8, 15.6]	0.900	*N* = 225 (Low)	*N* = 21 (Moderate)

CI, confidence interval [lower limit, upper limit]; MCV, mean coefficient of variation; ICC, intraclass correlation; MRD, mean relative difference.

a *N* = 25.

b *N* = 22 (three subjects completed only two visits).

c Reproducibility rating for 10% detection, power = 0.9, and significance = 0.05.

**Table 6:** Consistency of WM blood flow as measured by pCASL

pCASL WM	V1 Mean [95% CI^a^]	V2 Mean [95% CI^a^]	V3 Mean [95% CI^b^]	MCV (%) [95% CI]	MRD (%) [95% CI]	ICC	Rating 3%^c^	Rating 10%^c^
Average	7.39 [6.67, 8.12]	7.49 [6.70, 8.28]	7.32 [6.57, 8.08]	4.7 [3.2, 6.1]	6.3 [4.1, 8.1]	0.982	*N* = 49 (Low)	*N* = 6 (High)
Genu	11.7 [10.5, 12.8]	11.7 [10.5, 12.9]	11.3 [9.98, 12.6]	8.3 [6.3, 10.4]	10.7 [8.2, 13.1]	0.956	*N* = 136 (Low)	*N* = 13 (High)
Body	12.5 [11.3, 13.8]	13.0 [11.6, 14.4]	12.4 [10.9, 14.0]	8.6 [7.0, 10.2]	11.1 [9.1, 13.2]	0.961	*N* = 159 (Low)	*N* = 15 (High)
Splenium	9.84 [8.83, 10.9]	9.90 [8.71, 11.1]	9.37 [8.07, 10.7]	9.8 [7.3, 12.3]	12.1 [9.3, 15.0]	0.950	*N* = 200 (Low)	*N* = 19 (High)
Fornix	6.95 [5.84, 8.07]	7.49 [6.51, 8.47]	6.80 [5.91, 7.69]	19.3 [14.1, 24.6]	27.1 [18.5, 35.8]	0.872	*N* = 757 (Low)	*N* = 69 (Low)
Corticospinal	3.38 [2.31, 4.46]	3.74 [2.61, 4.87]	3.19 [2.11, 4.27]	47.4 [33.3, 61.5]	76.3 [40.1, 112.6]	0.900	*N* = 2690 (Low)	*N* = 243 (Low)
Internal capsule	10.9 [9.63, 12.1]	11.3 [9.99, 12.7]	10.5 [9.12, 12.0]	11.4 [9.2, 13.6]	15.3 [12.1, 18.6]	0.946	*N* = 263 (Low)	*N* = 25 (Moderate)
Corona radiata	9.33 [8.23, 10.4]	9.71 [8.53, 10.9]	9.05 [7.87, 10.2]	9.8 [7.8, 11.8]	12.7 [10.1, 15.4]	0.947	*N* = 219 (Low)	*N* = 21 (Moderate)
Thalamic radiation	9.16 [8.04, 10.3]	9.49 [8.28, 10.7]	8.91 [7.68, 10.1]	10.6 [7.8, 13.3]	13.5 [9.7, 17.3]	0.949	*N* = 241 (Low)	*N* = 23 (Moderate)
Sagittal striatum	9.34 [8.28, 10.4]	9.94 [8.73, 11.2]	8.77 [7.73, 9.81]	10.2 [7.4, 12.9]	13.1 [9.7, 17.4]	0.949	*N* = 229 (Low)	*N* = 22 (Moderate)
External capsule	11.9 [10.8, 13.0]	12.5 [11.3, 13.7]	11.4 [10.2, 12.7]	10.4 [7.9, 13.0]	13.4 [10.1, 16.7]	0.926	*N* = 223 (Low)	*N* = 21 (Moderate)
Cingulum	17.1 [15.3, 19.0]	17.4 [15.6, 19.3]	16.5 [14.2, 18.7]	10.0 [7.2, 12.7]	13.3 [9.3, 17.4]	0.953	*N* = 203 (Low)	*N* = 19 (High)
Superior longitudinal fasciculus	11.2 [9.93, 12.5]	11.7 [10.3, 13.2]	11.0 [9.49, 12.5]	10.9 [8.1, 13.7]	14.4 [10.2, 18.6]	0.935	*N* = 249 (Low)	*N* = 25 (Moderate)
Fronto‐occipital	10.2 [8.82, 11.5]	10.7 [9.30, 12.0]	10.1 [8.78, 11.5]	13.3 [10.5, 16.1]	17.9 [13.1, 22.6]	0.919	*N* = 382 (Low)	*N* = 35 (Moderate)

CI, confidence interval [lower limit, upper limit]; MCV, mean coefficient of variation; ICC, intraclass correlation; MRD, mean relative difference.

a *N* = 25.

b *N* = 22 (three subjects only completed two visits), mean values for three visits in units of ml/100 g/min.

c Reproducibility rating for 10% detection, power = 0.9, and significance = 0.05.

**Table 7:** Consistency of proton magnetic resonance spectroscopy

Metabolite	V1 Mean [95% CI^a^]	V2 Mean [95% CI^a^]	V3 Mean [95% CI^b^]	MCV (%) [95% CI]	MRD (%) [95% CI]	ICC	Rating (3%)^c^	Rating (10%)^c^
*TE135 frontal lobes WM*								
Mean tCho	1.91 [1.81, 2.01]	1.86 [1.78, 1.94]	1.89 [1.79, 1.99]	3.9 [3.1, 4.7]	5.0 [3.9, 6.0]	0.962	*N* = 35 (Moderate)	*N* = 4 (High)
Mean tNAA	11.0 [10.6, 11.4]	11.0 [10.7,11.4]	10.9 [10.6, 11.3]	2.8 [1.8, 3.9]	3.7 [2.3, 5.1]	0.914	*N* = 19 (High)	*N* = 3 (High)
Mean tCr	5.05 [4.89, 5.21]	5.0 [4.80, 5.12]	5.03 [4.87, 5.19]	4.1 [3.1, 5.1]	5.3 [4.0, 6.7]	0.851	*N* = 38 (Moderate)	*N* = 5 (High)
*TE30 frontal lobes WM*								
Frontal mean Glu	8.16 [7.73, 8.60]	7.74 [7.41, 8.08]	7.91 [7.45, 8.36]	7.8 [6.3, 9.3]	10.1 [7.9, 12.2]	0.816	*N* = 141 (Low)	*N* = 14 (High)
Frontal mean tCho	2.27 [2.14, 2.41]	2.23 [2.13, 2.32]	2.27 [2.12, 2.42]	6.4 [4.6, 8.2]	8.3 [5.8, 10.7]	0.886	*N* = 91 (Low)	*N* = 9 (High)
Frontal mean tNAA	9.98 [9.62, 10.3]	9.92 [9.61, 10.23]	9.86 [9.51, 10.2]	4.7 [2.9, 6.6]	6.1 [3.7, 8.4]	0.694	*N* = 51 (Low)	*N* = 6 (High)
Frontal mean mI	5.51 [5.24, 5.78]	5.34 [5.02, 5.66]	5.31 [4.99, 5.63]	8.2 [6.2, 10.2]	10.7 [8.1, 13.3]	0.745	*N* = 155 (Low)	*N* = 15 (High)
Frontal mean tCr	7.32 [7.06, 7.57]	7.01 [6.81, 7.22]	7.02 [6.75, 7.30]	6.1 [4.3, 7.9]	7.7 [5.3, 10.1]	0.565	*N* = 84 (Low)	*N* = 9 (High)
Frontal mean Glu+Gln	9.86 [9.30, 10.4]	9.59 [9.22, 9.95]	9.62 [9.10, 10.1]	7.2 [5.7, 8.6]	9.0 [7.0, 11.1]	0.818	*N* = 119 (Low)	*N* = 12 (High)
Frontal mean GSH	2.44 [2.28, 2.59]	2.36 [2.24, 2.49]	2.31 [2.10, 2.51]	11.3 [8.2, 14.5]	14.1 [10.6, 17.6]	0.696	*N* = 281 (Low)	*N* = 26 (Moderate)
*TE30 AC WM*								
AC Glu	13.3 [12.8, 13.7]	13.4 [13.1,13.7]	13.0 [12.6, 13.4]	4.2 [3.3, 5.1]	5.5 [4.2, 6.7]	0.763	*N* = 43 (Low)	*N* = 5 (High)
AC GSH	2.43 [2.32, 2.55]	2.46 [2.35, 2.58]	2.42 [2.33, 2.52]	6.1 [4.6, 7.6]	7.8 [5.9, 9.8]	0.798	*N* = 87 (Low)	*N* = 9 (High)
AC tCho	2.20 [2.08, 2.31]	2.23 [2.14, 2.31]	2.18 [2.07, 2.28]	4.8 [3.5, 6.2]	6.3 [4.4, 8.2]	0.879	*N* = 52 (Low)	*N* = 6 (High)
AC tNAA	11.4 [11.1, 11.6]	11.4 [11.2, 11.6]	11.4 [11.2, 11.6]	2.5 [1.8, 3.2]	3.2 [2.3, 4.1]	0.787	*N* = 15 (High)	*N* = 3 (High)
AC mI	6.65 [6.38, 6.91]	6.75 [6.53, 6.98]	6.62 [6.40, 6.85]	4.3 [3.2, 5.4]	5.6 [4.2, 6.9]	0.781	*N* = 44 (Low)	*N* = 6 (High)
AC tCr	10.4 [10.2, 10.7]	10.4 [10.2, 10.6]	10.2 [10.0, 10.4]	2.9 [2.2, 3.6]	3.8 [2.8, 4.7]	0.667	*N* = 21 (Moderate)	*N* = 3 (High)
AC Glu+Gln	15.1 [14.6, 15.6]	15.2 [14.8, 15.5]	15.0 [14.5, 15.5]	4.6 [3.6, 5.5]	5.9 [4.6, 7.2]	0.765	*N* = 51 (Low)	*N* = 4 (High)

AC, anterior cingulate; MRD, mean relative difference; CI, confidence interval [lower limit, upper limit]; MCV, mean coefficient of variation; Glu, glutamate; Glu+Gln, glutamate+glutamine; GSH, glutathione; GM, gray matter; ICC, intraclass correlation; mI, myo‐Inositol; Cr, total creatine; tCho, choline; tNAA, total *N* ‐acetylaspartate; WM, white matter.

a *N* = 25.

b *N* = 22 (three subjects only completed two visits), mean metabolites are in institutional units.

c Reproducibility rating for 10% detection, power = 0.9, and significance = 0.05.

(3) $$\text{MCV}=\frac{\sqrt{\frac{1}{2}\sum_{i=1}^{3}{\left(v_{i}-\overset{¯}{v}\right)}^{2}}}{\overset{¯}{v}}\times 100\;\text{where}\;\overset{¯}{v}=\frac{v_{1}+v_{2}+v_{3}}{3}$$

(4) $$\text{MRD}=\frac{|\frac{v_{1}-v_{2}}{v_{1}}|+|\frac{v_{2}-v_{3}}{v_{2}}|+|\frac{v_{3}-v_{1}}{v_{3}}|}{3}\times 100$$

(5) $$\text{ICC}=\frac{{\left(\sqrt{\frac{1}{2}\sum_{i=1}^{3}{\left(v_{i}-\overset{¯}{v}\right)}^{2}}\right)}^{2}}{{\left(\sqrt{\frac{1}{2}\sum_{i=1}^{3}{\left(v_{i}-\overset{¯}{v}\right)}^{2}}\right)}^{2}+{\left(\sqrt{\frac{1}{1}\sum_{i=1}^{2}{\left(v_{i}-\overset{¯}{v}\right)}^{2}}\right)}^{2}+{\left(\sqrt{\frac{1}{1}\sum_{i=2}^{3}{\left(v_{i}-\overset{¯}{v}\right)}^{2}}\right)}^{2}+{\left(\sqrt{\frac{1}{1}\sum_{i=1}^{3}{\left(v_{i}-\overset{¯}{v}\right)}^{2}}\right)}^{2}}$$

Finally, we calculated a “reproducibility rating” based on the variance observed for each trait across the three visits. This rating is based on the number of subjects per group needed to detect a 3% and 10% change for each measure calculated using a power analysis as detailed elsewhere (Iscan et al., 2015). The power analysis was performed under the following assumption: two‐group comparison with an equal number of subjects performed using a two‐tailed t‐test with the significance level set at *p* = .05 and a power of 0.90. We gave empiric rating of high reproducibility for measurements that required two groups of <20 subjects each. Medium and low reproducibility ratings were assigned for measurements that required two groups of 40 subjects and >40 subjects per group, respectively.

## 3 RESULTS

We separated measurements into structural and physiological. Structural measurements included cortical gray matter thickness, FLAIR WMH volume and count, DTI‐FA, and MBI. Physiological measurements included CBF and concentrations of neurochemicals. In general, structural measurements demonstrated greater consistency than physiological measurements (Tables 1, 2, 3, 4, 5, 6, 7).

MCV, MRD, and ICC for subcortical white matter hyperintensity volume/count on FLAIR showed better consistency compared to periependymal white matter hyperintensity volume and count in terms of higher ICCs (Table 1; ICC range 0.465–0.998). In terms of volume, MCVs and MRDs for subcortical and periependymal white matter hyperintensity volume were comparable. In terms of number of lesions, subcortical lesion count reproducibility was better than periependymal lesion count as evidenced by lower MCV and MRD values. Whole‐brain cortical gray matter thickness was highly consistent, while individual segments had more variability, with entorhinal, insula, and medial orbitofrontal being the least consistent in terms of ICC (Table 2; ICC range 0.747–0.987). All measurements were high or moderate on the 3% and high on the 10% reproducibility rating scale.

MCV, MRD, and ICC for whole‐brain global FA had excellent reproducibility while individual tracts varied in consistency (Table 3; ICC range 0.865–0.979). The least consistent tracts were the fornix, corticospinal, and fronto‐occipital as evidenced by the highest MCV and MRD values and lowest ICCs. MBI (commonly referred to as q‐space) was more consistent in the corpus callosum than anterior cingulate, with M_u_ more consistent than PDI (Table 4; ICC range 0.434–0.967). All measurements were high on the 3% and 10% reproducibility rating scale.

MCV, MRD, and ICC for whole‐brain gray matter pCASL were consistent, while individual segments varied, with greatest variability in the inferior temporal gyrus anterior, subcallosal cortex, cingulate gyrus anterior, parahippocampus gyrus anterior, and temporal fusiform cortex, posterior division (Table 5; ICC range 0.885–0.971). Whole‐brain white matter pCASL was also consistent, with again more variability in individual regions. The regions of greatest variability were the fornix and corticospinal (Table 6; ICC range 0.872–0.982). Most measurements were low on the 3% and 10% reproducibility rating scale with some exceptions. Gray matter average CBF was moderate at 10% while white matter was high at both 3% and 10%. Other white matter values had high or moderate reproducibility rating at 10% except for the FX, corticospinal, and fronto‐occipital.

For white matter TE135 spectroscopy, MCV and MRD were the lowest in tNAA (reflecting N‐acetylaspartate and N‐acetylaspartylglutamate) compared to tCho (reflecting glycerophosphocholine and phosphocholine) and tCr (reflecting creatine and phosphocreatine) (Table 7; ICC range 0.851–0.962). White matter TE30 for frontal lobe trended lower in consistency (Table 7; ICC range 0.565–0.886), with tCho being the most consistent and total creatine least consistent in terms of ICC. Again, tNAA had the lowest MCV and MRD and GSH had the highest MCV and MRD values. Gray matter TE30 for anterior cingulate metabolites (Table 7; ICC range 0.667–0.879) was similar, with total choline most consistent and total creatine least consistent in terms of ICCs, while tNAA had the lowest MCV and MRD values, whereas GSH had the highest values. All measurements were high on the 3% and 10% reproducibility rating scale except for the TE30 frontal lobe (GLU, mI, glutamate + glutamine (GLU+GLN)) and TE30 anterior cingulate (GLU+GLN).

## 4 DISCUSSION

Utilization of repeated MRI measurements for longitudinal studies of disease progression and treatment effects depends on the reproducibility of MRI measurements. The inherent technical and physiological variability in MRI measurements may contribute to measurement errors and interfere with detection of change due to advancing pathological or therapeutic changes. Multiple technical factors contribute to variability on repeat imaging, including variability in MRI scanners, sequences, MRI technicians, and MRI interpretation. No structural change over a short interval in a healthy cohort would be anticipated. Physiological variability, however, including activity level change, diurnal variation, or nutritional and/or alcohol intake, might impact measurements. Prior to interpreting the effect of a disease state, reproducibility or consistency must be known. The aim of this study of 25 healthy subjects is to provide reference data on intrasubject variability by controlling for these other factors, thus establishing a baseline power level to help with understanding the statistical significance of the observed changes.

This manuscript quantifies reproducibility and normal physiological variability for commonly used imaging and spectroscopic measurements. Previous efforts demonstrated high scan–rescan reproducibility of the neuroimaging included of the volumetric measurements for subcortical brain structures (Maclaren et al., 2014), cortical gray matter thickness (Dickerson et al., 2008; Han et al., 2006; Li et al., 2015), and diffusion tensor measurements (Acheson et al., 2017; Jovicich et al., 2014). Likewise, several prior studies quantified scan–rescan stability and reproducibility of the MRS measurements at 3T (Wellard, Briellmann, Jennings, & Jackson, 2005; Wijtenburg & Knight‐Scott, 2011; Wijtenburg et al., 2013). Our approach of three scanning sessions and tightly controlled methodological parameters provides for the opportunity to assess these measurements based on the normal physiological variance among them.

White matter hyperintensity quantification for subcortical lesion volume/count was highly reproducible. Similarly, periependymal white matter hyperintensity volume was reproducible, but count less so. Pulsation of ventricular cerebrospinal fluid and subject motion may cause artifacts, with partial volume averaging impeding accurate segmentation of small (<1 cm^3^) periependymal lesions (De Coene et al., 1992; Gawne‐Cain, Silver, Moseley, & Miller, 1997; Kates, Atkinson, & Brant‐Zawadzki, 1996). Subcortical lesions are unaffected by cerebral spinal fluid (CSF) pulsation artifacts and had higher ICC. We believe that higher variance in periependymal count measurements is secondary to these artifacts, making accurate identification of small periependymal lesions more challenging. This effect is further exaggerated by much smaller (3–5 times) number and volume of lesions in this healthy sample, compared to those reported in the general population (Kochunov et al., 2009, 2010), magnifying the effect of misidentifying even a single small lesion.

Overall whole‐brain average and regional cortical gray matter thickness and volumetric measurements showed excellent ICC and other measures of reproducibility that were consistent with other published results (Iscan et al., 2015; Liem et al., 2015; Yang et al., 2016). The cortical gray matter thickness of the entorhinal, insula, and medial orbitofrontal demonstrated lower reproducibility. These three cortical gray matter areas are located on the inferior frontal portion of the brain where susceptibility artifacts due to tissue–bone interface make the precise identification of boundaries more difficult. Therefore, caution should be recommended when interpreting cortical gray matter thickness findings from these areas. The power analyses estimates provided here showed a smaller number of subjects per group (*N *~ 10) than Liem (*N* = 40) (Liem et al., 2015) but similar to that provided by Iscan (*N* = 19) (Iscan et al., 2015). This is due to a difference in methodology. Our approach was based on the variance in the average gray matter (GM) thickness measurements that was also used by Iscan (Iscan et al., 2015). The power analysis by Liem and colleagues provided the number of subjects needed to detect the vertex‐specific difference in mean thickness by accounting for vertex‐wise variance across the surface.

Whole‐brain FA was highly reproducible, with individual tracts showing only slightly reduced reproducibility metrics than the whole‐brain average FA. The least consistency was observed in fornix (FX), corticospinal (CST), and superior fronto‐occipital (SFO) tracts. The lack of consistency on these three tracts can be explained by partial volume averaging and/or spatial misregistration and is similar to previous reports (Vollmar et al., 2010). The FX and CST are long, tubular white matter that passes through the areas with magnetic susceptibility and therefore prone to geometrical distortions. Our overall results are comparable to other reports. Jansen and colleagues performed two scans on 10 healthy subjects, noting median lobar DTI(FA) values for ICC of 0.75–0.86 (Jansen, Kooi, Kessels, Nicolay, & Backes, 2007). Bisdas and colleagues performed two scans separated by 2 weeks on 12 subjects, noting low individual FA tract MCV (0.4%–10%) (Bisdas, Bohning, Besenski, Nicholas, & Rumboldt, 2008). Vollmar and colleagues noted FA MCV of 0.8%–3.0% and ICC of 0.82–0.99 in nine subjects, with the whole‐brain average FA showing the smallest MCV (Vollmar et al., 2010). Veenith and colleagues in 22 subjects noted a mean ICC of 0.78% (0.56%–0.98%) and MCV of 0.69% (0.42%–0.99%) (Veenith et al., 2013). The regional pattern of reproducibility measurements was similar to that reported in Acheson et al. (2017). Future work evaluating these regions should take caution in interpreting any results localized to the CST and FX.

Measurements of the unrestricted water fraction *M*_u_ and permeability‐diffusivity index from the diffusion‐weighted data collected in the white matter of corpus callosum were highly reproducible. The same measurements performed in the anterior cingulate gray matter were more variable, suggesting tissue‐specific variance in normal physiology. There are two potential sources of variability in the anterior cingulate. The variance in diffusion‐based measurements is likely to be influenced by normal day‐to‐day physiological variability in the gray matter. The higher variance may also be due to methodological sources as the measurements from the dense and consistently oriented fibers of the corpus callosum may have greater reproducibility than the measurements from the cortical GM ribbon that is adjacent for WM and CSF. The tissue‐related difference in the reproducibility was likewise observed in the resting CBF as measured by pCASL. The whole‐brain average CBF in cerebral white matter showed higher reproducibility than in cerebral gray matter, while the anterior cingulate gyrus was lower. Our results are consistent with other reported studies. In eight participants, Wu and colleagues noted ICC/MCV values for gray matter of 0.926/4.67% and for white matter of 0.727/6.02%, with higher variability observed when examining individual regions (Wu, Lou, Wu, & Ma, 2014). In 12 healthy subjects scanned 1 week apart, Chen and colleagues noted gray matter ICC/MCV of 0.911/8.5% and white matter of 0.887/12.0%, with slightly greater variability found when examining individual regions (Chen, Wang, & Detre, 2011). Our results suggest pCASL can be utilized for comparison studies of whole‐brain and segment gray matter CBF. Additionally, while whole‐brain and corpus callosum white matter CBF is highly reproducible, other white matter tracts have greater variability.

Magnetic resonance spectroscopy assessment of neurochemical concentrations using a standard, clinical, long TE (TE = 135 ms) protocol demonstrated high reproducibility in frontal white matter for total choline, total N‐acetylaspartate, and total creatine. Our results are similar to other reports. Utilizing a 1.5T MR scanner, Li and colleagues reported an MCV of 8.3%–9.7% (Li, Babb, Soher, Maudsley, & Gonen, 2002), while Mullins and colleagues noted an MCV < 5%. (Mullins et al., 2003). Our results are similar to other reported series. In six subjects scanned twice using a 30 ms point resolved spectroscopy sequence, Mullins and colleagues observed comparable MCV (Mullins et al., 2003). In 10 healthy subjects scanned twice, Jansen and colleagues noted metabolic MCVs of 7.0%–20.4% and ICCs of 0.00–0.55 in the frontal and temporal lobes (Jansen et al., 2007). Wiebenga and colleagues noted a slightly higher MCV in 12 subjects with 6 months between TE30 scans (Wiebenga et al., 2014). The intrasubject higher MCVs reported by Ding and colleagues reflect his whole‐brain reproducibility metric rather than the small region of interest used in our study (Ding et al., 2015).

This study controlled the methodological parameters by using the same scanner, head coil, and MR operator. However, these conditions are unlikely to be maintained throughout the life of longer longitudinal or cross‐sectional studies where scanner upgrades, significant hardware changes such as changes head coil, and other methodological changes may be expected. To address these aspects of longitudinal studies, our group and others used two strategies to accommodate for methodological changes: collections of calibration data and use of meta‐ and mega‐analyses (Jahanshad et al., 2013; Kochunov et al., 2015; McGuire et al., 2014a). In the first approach, calibration data are collected before and after change to derive cross‐calibration parameters. This approach provides direct normalization and is the only appropriate method for longitudinal studies where different imaging points are collected on different scanners. The following challenges must be met: the calibration sample must match the constitution of the imaging sample and a sufficient number of calibration subjects must be collected to reduce uncertainty in calibration parameters. For instance, a more sensitive MRI coil provided higher (rise of 15%) FLAIR region counts with less dramatic change in volume (rise of 3%) due to ability to detect smaller lesions. Therefore, collecting FLAIR calibration datasets in a younger population with fewer and smaller lesions may have biased the calibration results. Likewise, while collecting 10 subjects was sufficient for FLAIR calibration, calibration of DTI data required 20 subjects to reduce uncertainty in FA measurements for smaller and more variable white matter tracts (Acheson et al., 2017). Alternatively, cross‐sectional and longitudinal studies with short interimaging periods can use statistical aggregation approaches that treat samples collected on different hardware as independent datasets. ENIGMA consortium has demonstrated the utility in meta‐ and mega‐analysis of quantitative neuroimaging data (Jahanshad et al., 2013; Kochunov et al., 2015).

This study measured the stability, reproducibility, and reliability in a healthy normal population of commonly utilized MRI modalities over an interval of 5 days while controlling for technical and physiological factors. We assessed the commonly used neuroimaging measures based on the ability to reproduce them and identified a subset of measurements with high variability due to methodological and/or physiological variances. We provide a power calculation‐based reproducibility rating and the number of subjects per group necessary to detect a 3% or 10% change. Caution should be exercised when reporting and interpreting outcomes based on these. Overall, this study reports high reliability for most of the neuroimaging measurements making them valuable for evaluation of disease states or treatment protocols.

## CONFLICT OF INTEREST

Dr. McGuire reports no disclosures. Dr. Wijtenburg reports no disclosures. Dr. Sherman reports no disclosures. Dr. Rowland serves on the editorial board of Schizophrenia Bulletin. Ms. Ryan reports no disclosures. Dr. Sladky reports no disclosures. Dr. Kochunov reports no disclosures.
